# *Albuca Bracteate* Polysaccharides Synergistically Enhance the Anti-Tumor Efficacy of 5-Fluorouracil Against Colorectal Cancer by Modulating β-Catenin Signaling and Intestinal Flora

**DOI:** 10.3389/fphar.2021.736627

**Published:** 2021-09-03

**Authors:** Xinyu Yuan, Jiao Xue, Yingxia Tan, Qingguo Yang, Ziyan Qin, Xiaodong Bao, Shengkai Li, Liangliang Pan, Ziqing Jiang, Yu Wang, Yongliang Lou, Lei Jiang, Jimei Du

**Affiliations:** ^1^ Wenzhou Key Laboratory of Sanitary Microbiology, Department of Microbiology and Immunology, School of Laboratory Medicine, Wenzhou Medical University, Wenzhou, China; ^2^ Central Laboratory, The First Affiliated Hospital of Wenzhou Medical University, Wenzhou, China

**Keywords:** polysaccharide, colorectal cancer, 5-fluorouracil, β-catenin, gut microbiota, short-chain fatty acids

## Abstract

The first-line treatment for colorectal cancer (CRC) is 5-fluorouracil (5-FU). However, the efficacy of this treatment is sometimes limited owing to chemoresistance as well as treatment-associated intestinal mucositis and other adverse events. Growing evidence suggests that certain phytochemicals have therapeutic and cancer-preventing properties. Further, the synergistic interactions between many such plant-derived products and chemotherapeutic drugs have been linked to improved therapeutic efficacy. Polysaccharides extracted from *Albuca bracteata* (*Thunb.*) *J.C.Manning and Goldblatt* (ABP) have been reported to exhibit anti-oxidant, anti-inflammatory, and anti-tumor properties. In this study, murine CRC cells (CT26) and a murine model of CRC were used to examine the anti-tumor properties of ABP and explore the mechanism underlying the synergistic interactions between ABP and 5-FU. Our results revealed that ABP could inhibit tumor cell proliferation, invasion, and migratory activity *in vitro and* inhibited tumor progression *in vivo* by suppressing β-catenin signaling. Additionally, treatment with a combination of ABP and 5-FU resulted in better outcomes than treatment with either agent alone. Moreover, this combination therapy resulted in the specific enrichment of *Ruminococcus*, *Anaerostipes*, *and Oscillospira* in the intestinal microbiota and increased fecal short-chain fatty acid (SCFA) levels (acetic acid, propionic acid, and butyric acid). The improvement in the intestinal microbiota and the increase in beneficial SCFAs contributed to enhanced therapeutic outcomes and reduced the adverse effects of 5-FU. Together, these data suggest that ABP exhibits anti-neoplastic activity and can effectively enhance the efficacy of 5-FU in CRC treatment. Therefore, further research on the application of ABP in the development of novel anti-tumor drugs and adjuvant compounds is warranted and could improve the outcomes of CRC patients.

## Introduction

Colorectal cancer (CRC) is the third most common cause of tumor-related death worldwide (Siegel et al., 2020; Siegel et al., 2021). Currently, the standard treatment for CRC consists of a combination of surgery and chemotherapy, with 5-fluorouracil (5-FU) serving as the first-line drug of choice in most cases (Shionome et al., 2013). Treatment with 5-FU restricts tumor cell growth and causes apoptosis *via* extensive DNA damage. However, many CRC patients eventually develop chemoresistance to 5-FU. In addition, 5-FU does not specifically target tumor cells and thus also causes damage to normal cells, resulting in potentially severe adverse effects. Moreover, 5-FU treatment can also alter the gut microbiota (Wardill et al., 2014; Hamouda et al., 2017), which influences intestinal barrier function and drives colon inflammation (Cai et al., 2021).

The gut microbiota plays a vital role in maintaining intestinal homeostasis, mucus barrier integrity, host immune responses, drug toxicity (Liu et al., 2020), and response to anti-cancer drugs (Karin et al., 2014). Polysaccharides, considered potential prebiotics, have beneficial effects on host intestinal health and may attenuate chemotherapy-induced intestinal mucositis (Shang et al., 2018; Gori et al., 2019). The anti-tumor effects of polysaccharides can be partially attributed to the regulation of the gut microbiota (Liu et al., 2019) and increases in fecal short-chain fatty acids (SCFAs) (Wang et al., 2017). Natural anti-tumor compounds with few or no adverse effects are appealing alternatives to adjuvant drugs in cancer treatment (Haq et al., 2019). Liu et al. found that tea polysaccharides can inhibit CT26 tumor cell proliferation and metastasis *via* the IL-6/STAT3 signaling pathway (Liu et al., 2018). Liang et al. found that *Ganoderma lucidum* polysaccharides can upregulate JNK expression *via* the MAPK pathway and thereby induce apoptosis in HCT-116 cells (Liang et al., 2014). Feng et al. found that *Atractylodes macrocephala* polysaccharides enhance anti-tumor immune responses by driving lymphocyte proliferation and elevating the production of IL-6, IFN-λ, and TNF-α (Feng et al., 2019). As such, these biocompatible phytochemicals represent promising adjuvant chemotherapeutic drugs for the treatment of various cancers (Liu, 2004).

*Albuca bracteata* (*Thunb.*) *J.C.Manning and Goldblatt* (AB), formerly known as *Ornithogalum caudatum* Aiton, is an evergreen perennial herb from the *Asparagaceae* family. It is native to southern Africa but has long been cultivated in China. AB is a herbal remedy for hepatitis, diabetes, parotitis, and several other diseases in both traditional Chinese medicine (TCM) and African folk medicine (Zhang et al., 2017). Molecular analyses have ascribed the bioactive properties of AB to a variety of saponins, polysaccharides, and flavonoids, which exhibit anti-neoplastic, anti-oxidant, anti-diabetic, and anti-inflammatory effects (Zhou et al., 2005). Chen et al. reported that AB has a relatively high content of polysaccharides, which can be effectively extracted using the ultrasonic extraction technique (Chen et al., 2012). AB polysaccharides (ABP) may be good candidates for anti-oxidant and immunostimulating agents in medicine and functional foods. However, few studies, with the exception of one demonstrated that ABP treatment suppresses murine S180 sarcoma growth, have focused on the specific anti-tumor properties of ABP. Moreover, to our knowledge, no study has explored the effect of ABP treatment in the context of gastrointestinal tumors.

β-catenin is a multifunctional oncogenic protein that plays an essential role in developmental processes (Clevers and Nusse, 2012). Abnormal activation of the Wnt/β-catenin pathway in the context of CRC results in excess accumulation of nuclear β-catenin, further driving tumor cell proliferation and malignancy and ultimately facilitating CRC development and progression (Reya and Clevers, 2005). Apart from its role in cell growth and adhesion, the activated canonical Wnt/β-catenin signaling pathway also promotes tumor bulking, recurrence, and resistance to chemotherapy in cancer stem cells (Reya and Clevers, 2005; Malanchi et al., 2008). High levels of β-catenin induce chemoresistance in CRC cells (Jiang et al., 2018; Uppada et al., 2018), and increased expression of β-catenin has been observed in drug-resistant CRC cells (He et al., 2018). Saito et al. found that β-catenin silencing is sufficient to sensitize CRC cells to chemotherapeutic treatment (Saifo et al., 2010). Accordingly, agents capable of inhibiting β-catenin could improve the clinical outcomes of cancer patients when used in combination with standard therapies.

Herein, we explored the potential therapeutic utility of ABP treatment and mechanisms underlying the potential anti-neoplastic synergy between 5-FU and ABP *in vivo* and *in vitro,* hoping to find a new strategy for CRC treatment. We determined that ABP can suppress the migratory, proliferative, and invasive activity of CRC cells by inhibiting the Wnt/β-catenin signaling pathway. Further, we found that ABP can enhance the ability of 5-FU to suppress CRC, likely by downregulating β-catenin, improving the overall composition of the intestinal microbiome, and augmenting SCFA levels. As such, our data suggest that ABP may be a promising anti-tumor agent and could serve as valuable adjuvant agents in chemotherapy for CRC.

## Materials and Methods

### ABP Preparation

ABP were extracted from AB bulbs and purified using a previously published protocol (Chen et al., 2012). The total carbohydrate, uronic acid, and protein content of the resultant extract was 92.58, 1.63, and 1.70%, respectively, and the average molecular weight was 18.3 kDa.

### Cell Culture

The CT26 murine cell line was purchased from the Type Culture Collection of the Chinese Academy of Sciences (Shanghai, China). Cells were cultured in RPMI-1640 medium containing 10% fetal bovine serum (Thermo Fisher Scientific, United States) at 37°C in a humidified incubator containing 5% CO2.

### CCK-8 Assay

A Cell Counting Kit-8 (CCK-8, Dojindo, Japan) assay was performed using the manufacturer’s instructions to assess cell viability (Wang et al., 2020). Briefly, at the end of the treatment period, 10 μL of CCK-8 solution was added to each well. After a 3-h incubation at 37°C, the absorbance at 450 nm was measured.

### EdU Uptake Assay

A BeyoClick™ EdU Cell Proliferation Kit with Alexa Fluor 488 (BeyoTime) was used according to the manufacturer’s instructions to monitor cell proliferation (Wang et al., 2019). Briefly, at the end of the treatment period, EdU (10 μM) was added to each well, and cells were incubated for 2 h at 37°C. The cells were then fixed with 4% paraformaldehyde, stained with an Apollo Dye Solution, and subjected to Hoechst 33,342 nuclear counterstaining. EdU-stained cells were visualized by using a fluorescence microscopy (Leica, Wetzlar, Germany), and images were processed using ImageJ software.

### Wound Healing and Transwell Assays

Wound healing assay was performed as previously described (Shen et al., 2020). CT26 cells were seeded into 6-well plates and cultured to confluence. The cell monolayer was scratched with a 100-μL pipette tip to generate a scratch wound. Recovery was allowed to occur for 24 h in serum-free RPMI-1640 containing the appropriate ABP concentrations, following which the cells were observed using an optical microscope. The migration area was analyzed under an inverted microscope using ImageJ software.

The transwell assay was also performed as described previously (Wang et al., 2020). For this assay, 24-well Boyden tissue culture plates (Corning, MA, United States) with an 8-μm-pore polycarbonate membrane were used. CT26 cells were seeded into the upper chambers, and 200 μL of serum-free RPMI-1640 was added (migration, 2×10^5^ cells, 24 h; invasion, 4×10^5^ cells, 48 h). The numbers of migrating or invading cells were counted in randomly chosen fields from duplicate chambers using an optical microscope. There were three replicates for each treatment, and each experiment was repeated thrice.

### Western Blot

Western blots were performed as described previously (Luo et al., 2021). RIPA buffer (Thermo Fisher, Waltham, MA, United States) was used to extract the total protein from samples. ECL Western Blotting Substrate (Thermo Fisher, Waltham, MA, United States) was used to detect protein bands on a Bio-Rad Gel Doc XR + system (Bio-Rad, United States). The primary antibodies used were as follows: anti-β-catenin (#ab68183, Abcam, United Kingdom), anti-Cyclin D1 (#ab40754, Abcam, United Kingdom), anti-c-Myc (#ab32072, Abcam, United Kingdom), anti-E-Cadherin (#3195, Cell Signaling Technology, United States), anti-Vimentin (#5741, Abcam, United Kingdom), anti-PCNA (#ab29, Abcam, United Kingdom), anti-COX-2 (#AF7003, Affinity Biosciences, United States), and anti-GAPDH (#ab181602, Abcam, United Kingdom).

### Animal Studies

Animal experiments were performed using protocols approved by the Institutional Animal Care and Use Committee of Wenzhou Medical University (wydw 2021-0224). CT26 cells are homologous tumors of BALB/c mice. According to reports in the literature (Li et al., 2019; Wan et al., 2020), the success rate of modeling of CT26 on BALB/c female mice was satisfied. BALB/c mice (female, 4–5 weeks old, Laboratory Animal Center, Wenzhou Medical University) were kept in a climate-controlled room of 20–22°C with a 12 h/12 h light-dark cycle. Each of the 24 mice received a subcutaneous injection of 5×10^5^ tumor cells in the right thigh. On day 5 after injection, they were randomized into four groups (n = 6/group): control, 5-FU, ABP, and 5-FU + ABP based on tumor size at the start of the treatment. ABP were administered to the latter two groups *via* oral gavage (0.5 mg/ml; 0.2 ml/mouse) once per day for 16 days, whereas the other two groups received distilled water. Further, groups 2 and 4 received 5-FU *via* intraperitoneal injection for 10 days (20 mg/kg/mouse on each day), whereas the other two groups received sterilized distilled water. The body weight and tumor growth (volume = 0.5 × length × [width]^2^) in all animals were measured every alternate day. At the end of interval period, tumors were immediately removed after the animal was sacrificed, and weighed, fixed, or kept at −80°C.

### 16S rRNA Gene Sequencing and Microbiome Analysis

After euthanasia at the end of treatment, mouse fecal samples were collected and preserved at −80°C. Part of samples were used for microbiome composition analysis. 16S rRNA gene amplicon sequencing was performed using Illumina sequencing technology as previously described (Peng et al., 2019). Briefly, bacterial DNA was extracted from mouse feces using an E. Z.N.A.^®^ soil DNA Kit (Omega Bio-Tek, Norcross, GA, United States) according to the manufacturer’s instructions. The V3–V4 hypervariable region of the *16 sRNA* gene was amplified using the primers 338F (5ʹ-ACT​CCT​ACG​GGA​GGC​AGC​A-3ʹ) and 806R (5ʹ-GGACTACHVGGGTWTCTAAT-3ʹ). Amplified fragments were mixed in equimolar amounts and subjected to paired 2 × 300 bp sequencing on an *Illumina MiSeq* system. The Atacama soil microbiome tutorial from Qiime2docs was used for bioinformatics analyses along with custom scripts (https://docs.qiime2.org/2019.1/).

### Analysis of SCFAs

Fecal SCFA levels at the end of the intervention period were measured using a Trace GC Ultra Gas Chromatograph coupled with an ISQ Mass Spectrometer (TRACE 1310-ISQ 7000, Thermo, MA, United States). Final data were normalized based on fecal weight.

### Immunohistochemical Staining

Immunohistochemistry (IHC) analyses were conducted as described earlier (Li et al., 2018). Briefly, tumor tissue was fixed in 4% paraformaldehyde and embedded in paraffin using standard techniques. Sections (5 μm) were obtained and stained with anti-Ki67 (#ab152976, Abcam, United Kingdom) antibodies. Images were acquired using a fluorescence microscope (DP80, Olympus, Japan).

### Statistical Analysis

Data are presented as means ± standard deviation (SD) or median (interquartile range). Normally distributed data showing variance homogeneity were analyzed by student’s t-tests, other data were analyzed *via* Kruskal–Wallis tests using SPSS 23.0 (SPSS Company, Inc., United States). *p* < 0.05 was the threshold for significance (n.s., not significant, *p < 0.05, **p < 0.01, and ***p < 0.001).

## Results

### ABP Treatment Suppresses CRC Cell Proliferation

**FIGURE 1:**
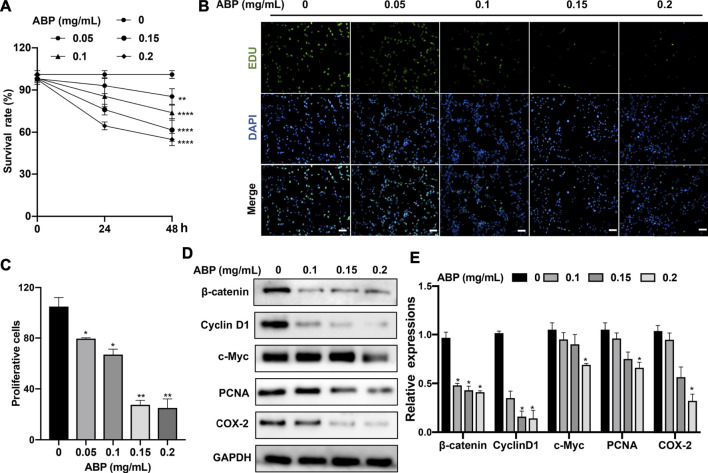
ABP treatment suppresses the proliferation of CRC cells. **(A)** CCK-8 assay examining the proliferation-suppressing effect of ABP on CT26 cells. **(B–C)** EdU uptake assay examining the proliferation-suppressing effect of ABP on CT26 cells. Scale bar = 50 μm (×200 magnification). **(D–E)** Western blot examining the effect of ABP on the protein levels of β-catenin, Cyclin D1, c-Myc, PCNA, COX-2, and GAPDH in CT26 cells. Compared with the untreated group, *p < 0.05, **p < 0.01.

To explore the effect of ABP treatment on CRC cells, we treated CT26 cells with a range of ABP concentrations and then explored their proliferation and viability through CCK-8 and EdU uptake assays. ABP treatment significantly impaired CT26 cell growth in a time- and dose-dependent manner (Figure 1A), and the EdU uptake assay yielded similar findings **(** Figures 1B,C **)**.

To further elucidate the mechanisms underlying the observed results, western blots were performed. They revealed that ABP treatment inhibited the expression of β-catenin, c-Myc, Cyclin D1, COX-2, and PCNA (Figures 1D,E). There is a corresponding change in *mRNA* levels (Supplementary Figure S1). The results thus suggested that ABP inhibit the growth of CRC cells *in vitro* by suppressing the Wnt/β-catenin pathway*.*

### ABP Treatment Suppresses the Migratory and Invasive Activity of CRC Cells

**FIGURE 2:**
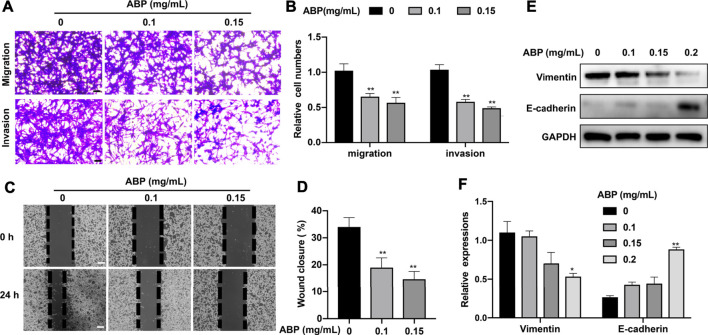
ABP treatment suppresses the migratory and invasive activity of CRC cells. **(A-B)** Transwell assay examining the negative effect of ABP on the migration and invasion of CT26 cells. Scale bar = 50 μm. **(C-D)** Wound healing assay examining the negative effect of ABP on the migration and invasion of CT26 cells. Scale bar = 250 μm. **(E-F)** Western blot examining the effect of ABP on the protein levels of Vimentin, E-cadherin, and GAPDH in CT26 cells. Compared with the untreated group, *p < 0.05, **p < 0.01.

The effects of ABP treatment on CRC cell migration and invasion were assessed using a transwell assay. Treatment with ABP at doses of 0.1 and 0.15 mg/ml resulted in a 38.3 ± 4.81% (*p* < 0.01) and 46.7 ± 6.24% (*p* < 0.01) decrease in the percentage of migratory cells, respectively. This was accompanied by a 42.6 ± 5.7% (*p* < 0.01) and 50.2 ± 4.1% (*p* < 0.01) decrease in the frequency of invasive cells (Figures 2A,B). Consistent with these results, wound healing assays showed that ABP treatment reduced the wound closure rate from 34 ± 3.4% in control samples to 19 ± 3.6% (0.1 mg/ml, *p* < 0.01) and 15 ± 3.1% (0.15 mg/ml, *p* < 0.01) in ABP treated ones (Figures 2C,D). Western blot analyses for epithelial–mesenchymal transition (EMT)-related proteins showed that ABP treatment suppressed Vimentin and enhanced E-cadherin expression in CT26 cells (Figures 2E,F).

### ABP Treatment Suppresses the Growth and Metastasis of CRC Cells by Downregulating β-Catenin

**FIGURE 3:**
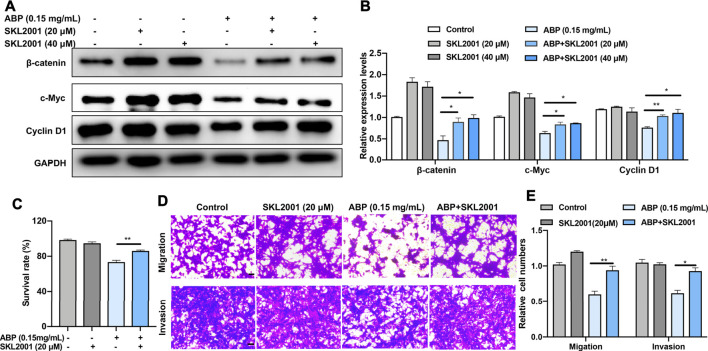
ABP treatment suppresses the growth and metastasis of CRC cells by downregulating β-catenin. **(A–B)** Western blot examining the effects of SKL 2001 (β-catenin activator) on β-catenin, c-Myc, and Cyclin D1 levels in CT26 cells treated with ABP. **(C)** CCK-8 assay examining the rescue effects of SKL2001 treatment on CT26 cells treated with ABP. **(D–E)** Transwell assay examining the rescue effects of SKL2001 treatment on CT26 cells treated with ABP. Scale bar = 50 μm. Compared with the ABP group, *p < 0.05, **p < 0.01.

Given our aforementioned results, which suggested that ABP suppress the expression of β-catenin and its related proteins, we speculated that β-catenin was an essential anti-tumor target of ABP in CT26 cells. To verify this hypothesis, we used an agonist of β-catenin, SKL 2001 (MCE, China), to manipulate β-catenin activity. We examined the effects of ABP treatment on CT26 cells with or without SKL2001 *in vitro* and analyzed subsequently. Results indicated that SKL2001 reversed the ABP-induced β-catenin downregulation (Figures 3A,B) and partially attenuated the ABP-induced inhibition of CRC cell proliferation (Figure 3C, *p* < 0.01), SKL2001 also evidently reversed the inhibitory effect of ABP on tumor cell migration (*p* < 0.01) and invasion (*p* < 0.05) (Figures 3D,E). These data demonstrated that ABP act as β-catenin inhibitors and inhibit the migration, invasion, and growth of CRC cells, at least in part, by downregulating β-catenin.

### ABP and 5-FU Exhibit Synergistic Anti-Tumor Efficacy Against CT26 Cells

**FIGURE 4:**
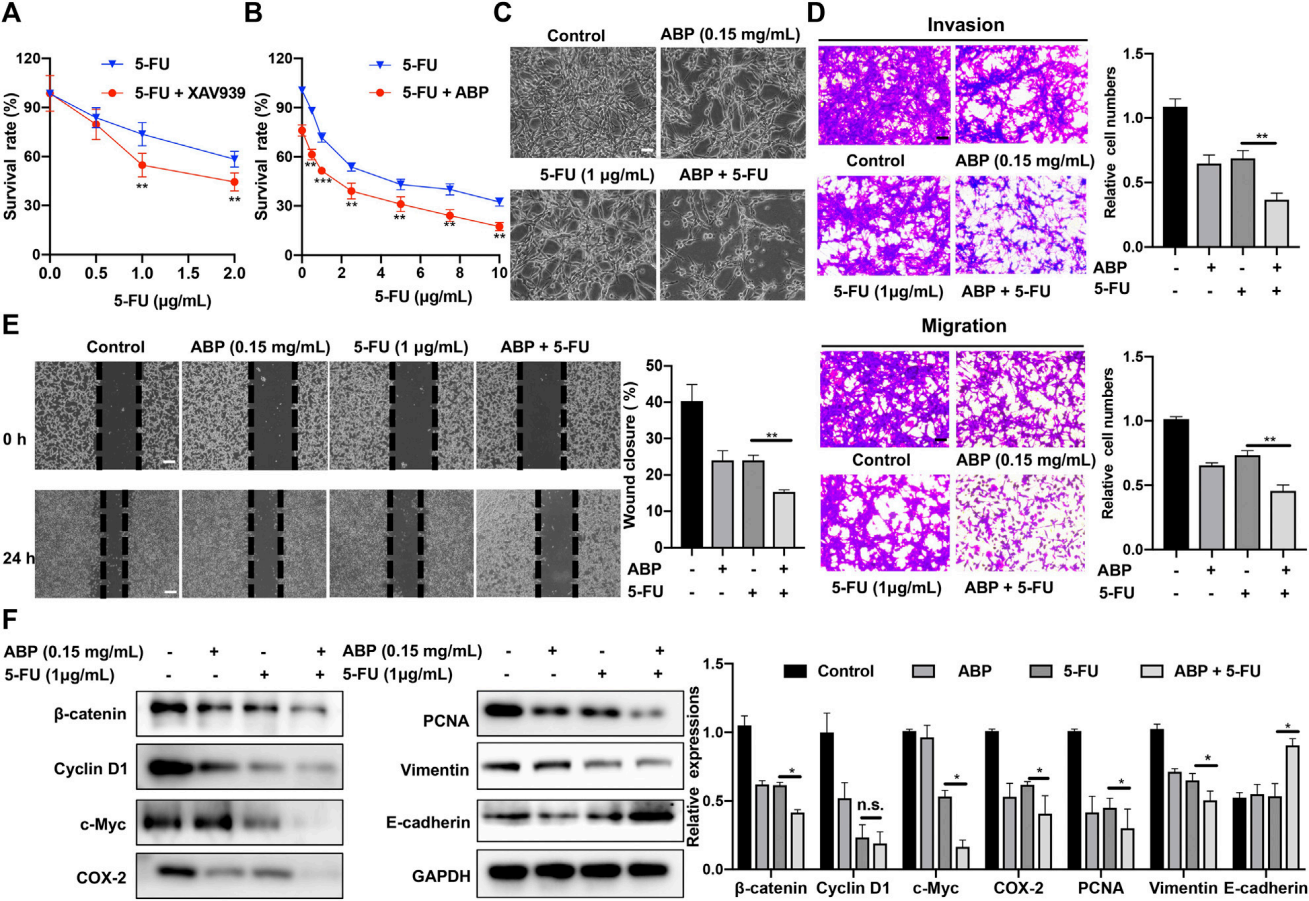
ABP enhances the anti-tumor efficacy of 5-FU against CT26 cells. **(A-B)** CCK-8 assay examining the effects of XAV939 (β-catenin inhibitor) and ABP on the anti-tumor efficacy of 5-FU against CT26 cells. **(C)** Cellular morphology of CT26 cells treated with ABP, 5-FU, and ABP + 5-FU for 24 h. Scale bar = 50 μm. **(D)** Transwell assays examining the enhancing effect of ABP on the anti-tumor efficacy of 5-FU against CT26 cells. Scale bar = 50 μm. **(E)** Wound healing assay examining the enhancing effect of ABP on the anti-tumor efficacy of 5-FU against CT26 cells. Scale bar = 250 μm. **(F)** Western blot examining the protein levels of β-catenin, Cyclin D1, c-Myc, COX-2, PCNA, Vimentin, E-cadherin, an GAPDH in CT26 cells treated with ABP and 5-FU. Compared with the 5-FU group, *p < 0.05, **p < 0.01, n. s., no significance.

A β-catenin inhibitor, XAV939, was used to assess the role of β-catenin in the cellular response to 5-FU. Subsequent analysis indicated that at doses of 1 μg/ml (*p* < 0.01) and 2 μg/ml (Figure 4A, *p* < 0.01), XAV939 enhances the cytotoxic effects of 5-FU on CT26 cells. We also used 2 *siRNAs* specific for β-catenin to knock down β-catenin expression in CT26 cells (Supplementry Figure S2). This result indicated that decreased β-catenin expression might enhance the chemotherapeutic efficacy of 5-FU. We then explored the combined effects of ABP (0.15 mg/ml) and 5-FU (0–10 μg/ml) on CT26 cells. CCK-8 assays showed that the survival rate in the combination treatment group was significantly lower than those in the ABP or 5-FU treatment group alone (Figures 4B,C). The effects of drug combinations were checked by using online software SynergyFinder (https://synergyfinder.org/) (Ianevski et al., 2020) and Compusyn (http://www.combosyn.com/) (Chou, 2010). The HSA synergy score (16.05), the Loewe synergy score (10.67), and the combination index (CI) (0.7 to 1.0) indicated that ABP and 5-FU had a slight synergistic effect *in vitro* (Supplementary Figure S3).

The effects of combination treatment on metastasis ability of CT26 cells were also evaluated by Transwell assays. Compared with 5-FU alone, the combination treatment significantly decreased migratory and invasive abilities by 38.3 ± 0.8% (*p* < 0.01) and 47.3 ± 5.3% (*p* < 0.01), respectively (Figure 4D). Furthermore, the combination treatment reduced the wound closure rate from 24 ± 2.5% of 5-FU alone to 14 ± 1.6% (Figure 4E, *p* < 0.01). Western blots showed that the expression levels of β-catenin, Cyclin D1, c-Myc, COX-2, PCNA, and Vimentin in the combination treatment cells were all lower than those in 5-FU alone, while the expression of E-cadherin was higher (Figure 4F). These data suggested that ABP had a synergistic effect with 5-FU on CRC tumor cell growth, migration, and invasion.

### ABP and 5-FU Exhibit Synergistic Anti-Tumor Efficacy in Tumor-Bearing Mice

**FIGURE 5:**
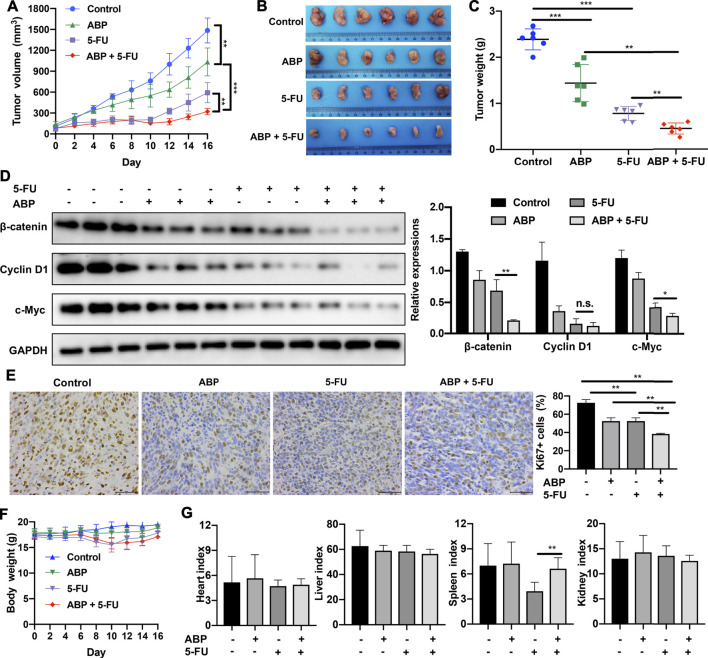
ABP exhibits synergistic anti-tumor effects when administered in combination with 5-FU in mice bearing CT26 tumors. **(A)** Tumor volumes measured at the indicated time points. Data are shown as mean ± SD (n = 6 mice at each time point). **(B)** Image of tumors resected from sacrificed mice at the end of the treatment period. **(C)** Tumor weights measured after surgical tumor removal, represented as mean ± SD. **(D)** Western blot examining the levels of β-catenin, Cyclin D1, and c-Myc in subcutaneous tumors, with GAPDH used as the internal control. **(E)** Images of Ki67 immunohistochemical staining (× 400) in tumor cells and quantification of Ki67-positive tumor cells. Scale bar = 50 μm. **(F)** Body weights measured every 2 days. **(G)** Organ indexes calculated as the ratio of organ weight (mg) to body weight (g) at the end of the treatment period. Data are means ± SD (*n* = 6). *p < 0.05, **p < 0.01, ***p < 0.001.

The anti-tumor efficacy of ABP *in vivo* was assessed in a BALB/c mouse model of subcutaneous CRC. ABP treatment significantly impaired tumor growth (Figures 5A–C); the average tumor weight in the ABP group (1.440 ± 0.401 g) was significantly lower than that in the control group (2.389 ± 0.228 g, *p* < 0.001) (Figures 5B,C). Further, the average tumor weight in the combination treatment group (0.449 ± 0.119 g) was also much lower than that in the 5-FU treatment group (0.781 ± 0.151 g, *p* < 0.01) (Figures 5B,C).

Western blots showed that ABP treatment reduced the expression levels of β-catenin, Cyclin D1, and c-Myc in tumor tissue samples (Figure 5D). IHC staining showed that ABP treatment reduced Ki-67 expression in tumor cells (Figure 5E). Significant body weight losses were observed in groups treated by 5-FU (Figure 5F, *p* < 0.01), but there was no statistical difference between the 5-FU and the combination group. The 5-FU group had the lowest spleen-to-body weight ratio (3.936 ± 1.071) and the combination treatment group had a significantly higher one (6.626 ± 1.311) (Figure 5G, *p* < 0.01). These data suggested that ABP exerted anti-tumor effects on CT26 tumor-bearing mice when used alone and provided synergistic anti-tumor effects when combined with 5-FU.

### ABP and 5-FU Treatment Improves the Composition of the Intestinal Flora in Mouse Models of CRC

**FIGURE 6:**
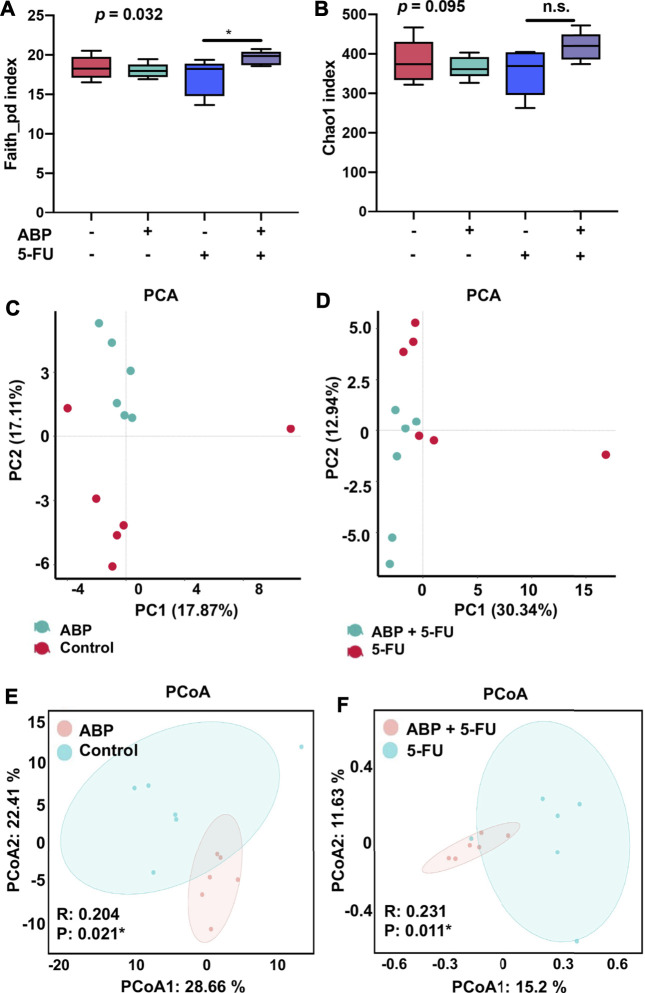
ABP treatment alters the abundance and diversity of gut microbes in mice bearing CT26 tumors. **(A-B)** Analysis of alpha diversity; **(A)** Faith_pd index. **(B)** chao1 index. **(C-F)** Analysis of beta diversity. **(C-D)** Principal components analysis (PCA). **(E-F)** principal coordination analysis (PCoA) (n = 6 mice per group).

**FIGURE 7:**
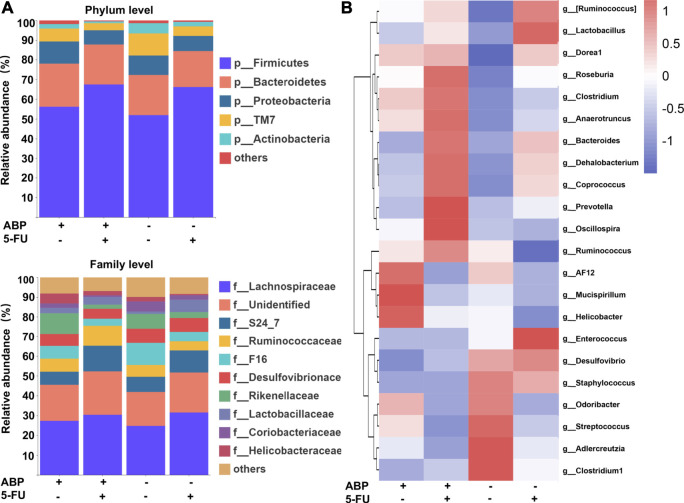
ABP treatment improves the composition of the intestinal microflora. **(** **A** **)** Bar plots of bacterial taxa present in the feces at the phylum and family levels based on relative abundance. **(** **B** **)** Heatmap of bacterial taxa present in the feces at the genus level based on relative abundance.

To explore whether the anti-tumor effects of ABP were associated with changes in intestinal flora, 16S rRNA gene sequencing of fecal flora was performed after treatment, and the species diversity was evaluated at the operational taxonomic unit (OUT) level. The combination treatment group had a higher alpha diversity than the 5-FU alone group (faith_pd index [*p* = 0.032], chao1 index [*p* = 0.095]) (Figures 6A,B, Supplementary Table S1), suggesting that the combination treatment improved the richness of intestinal flora. Principal component analysis (Figures 6C,D) and principal coordinates analysis (Figures 6E,F) showed clear differences between the control and ABP groups (R = 0.204, *p* = 0.021) and between the combination and 5-FU groups (R = 0.231, *p* = 0.011), indicating that ABP treatment significantly affected microbial composition. The composition of intestinal flora at the phylum and family level were shown in Figure 7A (Supplementary Table S2). To clarify the specific changes in the microbial taxa, we further analyzed the relative abundance of 22 predominant genera in the four groups. The results showed that at the genus level, the combination treatment group had significantly higher relative abundance of *Roseburia, Clostridium, Anaerotruncus, Bacteroides, Dehalobacterium, Corprococcus, Prevotella* and *Oscillospira* (Figure 7B), while the 5-FU treatment group had a highest level of *Enterococcus* and *Lactobaccilus.*

**FIGURE 8:**
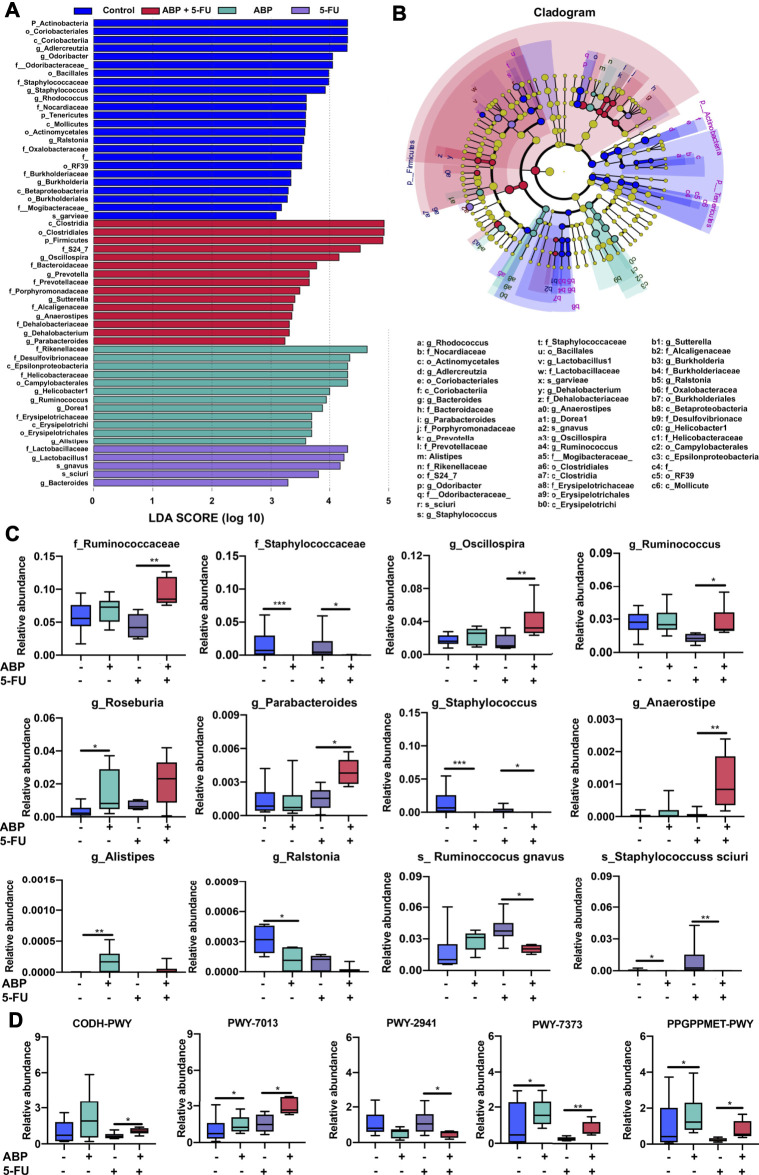
ABP treatment leads to the enrichment of beneficial bacteria and influences metabolic pathways. **(** **A-B** **)** Bacterial taxa differences among the four groups, observed using LEfSe analysis. **(C)** Box plots demonstrating the characteristic bacteria at the family, genus, and species levels. **(D)** Difference in functional metabolic pathways among the groups. *p < 0.05, **p < 0.01, ***p < 0.001.

To further identify microbial taxa that serve as biomarkers for different groups, we performed liner discriminate analysis (LDA) and effect size measurements (LEfSe). Biomarkers for each group ranked by effect size (*p* = 0.05, LDA score > 2) were shown in Figures 8A,B. To further clarify the taxa modulated by ABP, the Kruskal–Wallis test was applied to analyze the difference among the four groups. Compared with the 5-FU group, the combination treatment group harbored a higher relative abundance of some SCFA-producing bacteria (*f_Ruminococcaceae, g_Ruminococcus, g_Anaerostipes, g_Oscillospira, g_Parabacteroides*) and a lower relative abundance of certain potentially pathogenic, inflammation-promoting bacteria (*f_Staphylococcus, g_Staphylococcus, s_Staphylococcus sciuri, s_ Ruminoccocus gnavus*). Moreover, compared with the control group, the ABP treatment groups had a higher relative abundance of some SCFA-producing bacteria (*g_Alistipes, g_Roseburia*) and a lower abundance of potentially pathogenic bacteria (*g_Ralstonia, g_Staphylococcus, s_Staphylococcus sciuri*) (Figure 8C, Supplementary Table S3).

Compared to the 5-FU group, the relative abundance of bacteria involved in the PWY-7373 (superpathway of demethylmenaquinol-6 biosynthesis II), PWY-7013 (L-1,2-propanediol degradation), PPGPPMET-PWY (ppGpp biosynthesis), and CODH-PWY (reductive acetyl coenzyme A pathway) pathways was higher. In contrast, the PWY-2941 (L-lysine biosynthesis II) pathway was lower in the combination treatment groups than in the 5-FU group (Figure 8D, Supplementary Table S4). These data suggested that ABP treatment may ameliorate the dysbiosis induced by 5-FU, and combined treatment could enhance the therapeutic efficacy of 5-FU in part by increasing the levels of beneficial bacteria and improving metabolite composition.

### ABP Treatment Improves the Fecal SCFA Levels in Mouse Models of CRC

**FIGURE 9:**
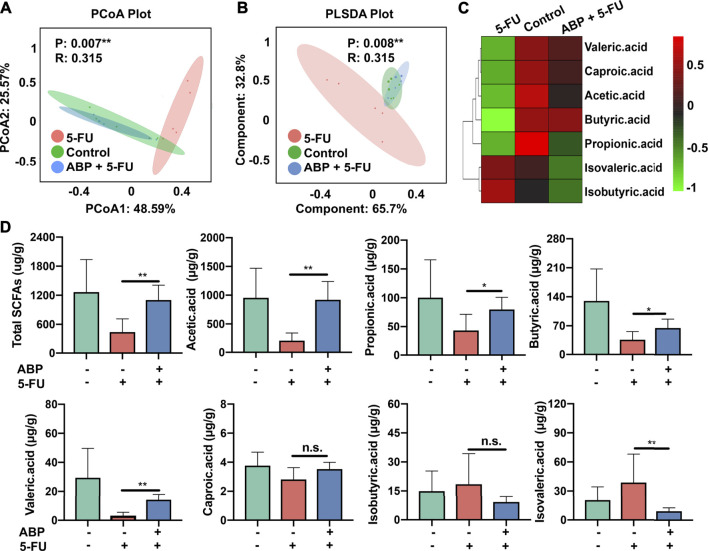
ABP treatment improves the concentrations of fecal short-chain fatty acids in CRC model mice. **(A-B)** PCoA and PLS-DA analysis. **(C-D)** Heatmap and Bar plots for fecal SCFA content. *p < 0.05, ** represents *p* < 0.01.

To elucidate the positive effects of ABP treatment on the metabolic activity of the intestinal flora, the concentrations of fecal SCFAs were measured using gas chromatography–mass spectrometry (GC–MS). PCoA and PLSDA analysis indicated that 5-FU treatment resulted in a distinct fecal SCFA composition profile (Figures 9A,B). A heat map was generated to visualize the data more intuitively. As shown in Figure 9C, the concentration of each beneficial SCFA in the 5-FU treatment group was lower than that of the other two groups. From the standpoint of positive impact, the combination treatment elevated all beneficial SCFA, especially the butyric acid (Figure 9C). The concentration of acetic acid (*p* < 0.01), propionic acid (*p* < 0.05), butyric acid (*p* < 0.05), valeric acid (*p* < 0.01), and caproic acid (*p* = 0.13) were higher in the combination treatment group than that in the 5-FU treatment group. In contrast, concentration of isovaleric acid (*p* < 0.01) and isobutyric acid (*p* = 0.23) were higher in the 5-FU treatment group (Figures 9C,D, Supplementary Table S5). These data suggested that 5-FU depleted most beneficial SCFAs, and ABP treatment could elevate the levels of beneficial SCFAs in the feces of CT26 tumor-bearing mice.

## Discussion

CRC is the second leading cause of cancer-related death in the United States and the third most common cancer worldwide. The use of 5-FU has been a standard approach for CRC treatment since the 1950s (Cho et al., 2020), and 5-FU-based chemotherapy remains the predominant approach for CRC, especially metastatic CRC (Van Cutsem et al., 2016). However, in addition to severe immunosuppressive effects and drug toxicity, 5-FU treatment also leads to primary and secondary chemoresistance (Xie N. et al., 2012). Given the enormous damage caused by cancer and the continuous emergence of chemoresistance, novel drugs and anti-tumor treatments are required. Further, the use of conventional chemotherapeutic agents in combination with natural compounds to provide synergistic anti-tumor effects could also be vastly helpful.

Natural products have served as important anti-tumor agents and have been developed for clinical use (Zi et al., 2019). Moreover, anti-tumor compounds with few or no adverse effects hold promise as drug alternatives or adjuvant agents in cancer treatment (Haq et al., 2019). Many plant-derived compounds such as *tanshinones*, curcumin, and Ganoderma lucidum polysaccharides have drawn great attention because of their broad-spectrum anti-tumor effects and high efficacy (Liang et al., 2014; Kaya et al., 2019; Fu et al., 2020).

Owing to their broad array of anti-inflammatory, anti-neoplastic, anti-oxidant, anti-bacterial, and anti-diabetic properties, plant polysaccharides have been the focus of intensive research (Ji et al., 2018). AB, a herbal remedy for hepatitis, diabetes, and many types of cancers (Zhang et al., 2017), has been used in TCM in some regions of China for several decades. Phytochemical examinations have confirmed that AB has broad bioactive properties owing to the various saponins, polysaccharides, flavonoids, and cholestane glycosides it contains (Zhou et al., 2005; Iguchi et al., 2017). Considering that TCM preparations of AB are generally decoctions and are administered orally, and given the high levels of potentially prebiotic polysaccharides AB contains (Chen et al., 2012), we speculated that ABP could contribute to the anti-tumor effects of AB. On testing this hypothesis, we found that ABP treatment effectively inhibits the growth, migration, and invasion of CRC cells *in vitro* and suppresses subcutaneous tumor growth *in vivo*.

CRC development is a complex, multi-step process, involving abnormalities in the Wnt/β-catenin, Hedgehog, Notch, TGF-β, Jak-STAT, and Ras-Raf-MAPK pathways (Calvert and Frucht, 2002). Aberrant Wnt/β-catenin pathway activation is evident in an estimated 90% of CRC tumors, and this pathway is therefore thought to play a central role in the development and malignant growth of such tumors (Kimelman and Xu, 2006). Abnormal activation of the classical Wnt signaling pathway results in high levels of β-catenin, driving EMT induction and cancer stem cell development, thereby enhancing the overall malignancy of CRC tumors (Ghahhari and Babashah, 2015). Cyclin D1 and c-Myc are downstream members of the β-catenin signaling pathway, and their overexpression results in enhanced proliferation and malignant transformation (Hsieh et al., 2012; Lin et al., 2015). Further, high c-Myc expression is also associated with poorer long-term outcomes in CRC patients (Matsushita et al., 2006). The EMT process is tightly linked to tumor cell invasion and metastasis, and it is characterized by E-cadherin downregulation and elevated N-cadherin and Vimentin expression (Vu and Datta, 2017).

We found that ABP treatment suppresses the expression of β-Catenin and related proteins, including c-Myc, Cyclin D1, PCNA, COX-2, and Vimentin, and elevates E-cadherin levels in a dose-dependent manner. β-catenin is crucial for Wnt/β-catenin signaling. Given that SKL2001 reverses ABP-induced β-catenin downregulation and cell proliferation, we concluded that β-Catenin was a crucial target of ABP in CT26 cells and suppressed the proliferative ability and malignancy of these cells at least partially by downregulating β-catenin and altering the expression of downstream genes.

Chemoresistance to 5-FU remains a major obstacle in CRC treatment. Drebber et al. reported that CRC patients with high levels of β-catenin show low sensitivity to 5-FU treatment (Drebber et al., 2011). In line with prior reports (Xie J. et al., 2012; Regmi et al., 2015), in our study, treatment with the β-catenin inhibitor XAV939 confirmed that the suppression of β-catenin signaling was sufficient to enhance the sensitivity of CRC cells to 5-FU. We further explored the effect of combined treatment with ABP and 5-FU on CT26 cells. The *in vitro* results showed that combined treatment provided better anti-tumor efficacy than ABP or 5-FU treatment alone. Further analysis indicated that ABP and 5-FU showed minor synergistic effects (Supplementary Figure S3).

Given the prebiotic potential of polysaccharides, we speculated that the efficacy of combination treatment would be better *in vivo*. We found that in a murine model of CRC, the anti-tumor efficacy of combination treatment was much better than that of single-agent treatment with 5-FU or ABP. Further, the spleen index was higher in the combination treatment group than in 5-FU-treated mice (Figures 5B,C,G). Moreover, a fascinating prolongation of the suppression period (4 days) was observed in the combination group (Figure 5A), while tumor proliferation resumed soon after the cessation of chemotherapy in the 5-FU-treated group.

To reveal the multiple mechanisms underlying the effects of ABP on CRC *in vivo*, western blot analyses were performed. We examined changes in proteins involved in the Wnt/β-catenin signaling pathway, and the results corroborated our *in vitro* findings.

Various factors contribute to CRC oncogenesis, including diet, drug intake, and environmental conditions, and all of these can shape the composition, function, and metabolite production capabilities of the intestinal microbiota (Wang et al., 2018). Billions of bacteria reside within the human colon, where they serve as essential bridges between the host and specific drug responses and functional nutrients, contributing to both normal homeostasis and dysbiosis-associated pathological conditions such as inflammatory bowel disease (Backhed et al., 2005; Raman et al., 2013).

Polysaccharides improve immune responses and chemosensitivity in cancer patients (Yin et al., 2015), and the anti-tumor effects of polysaccharides are partially due to their regulation of gut microbiota and promotion of fecal SCFA levels (Wang et al., 2017). In the colon, SCFAs serve as an essential energy source for epithelial cells and can regulate key inflammatory processes within the colon to maintain normal host physiology (Kim et al., 2014; Schulz et al., 2014; Yang et al., 2020). Hence, we analyzed the fecal microbiome composition and fecal SCFA concentration to clarify the anti-tumor functions of ABP. *16S rRNA* sequencing revealed that the genera *Ruminococcus, Anaerostipes, Oscillospira, Alistipes,* and *Roseburia* were enriched in fecal samples from the ABP treatment group. These bacteria are potential producers of SCFAs (Gophna et al., 2017; Kasahara et al., 2018; La Reau and Suen, 2018; Markowiak-Kopec and Slizewska, 2020; Parker et al., 2020). Data also indicated that ABP inhibited the growth of certain pathogenic inflammation-promoting bacteria (*g_Staphylococcus, s_Staphylococcus sciuri, s_-Ruminoccocus gnavus*). It was clear that ABP treatment ameliorated the dysbiosis induced by 5-FU and improved the intestinal flora composition in CRC model mice to a certain extent.

Butyric acid is a crucial SCFA in the intestinal tract, and it can kill CRC cells and inhibit their migration (Wang et al., 2020). GC–MS results revealed that ABP could increase the levels of some beneficial SCFAs (acetic acid, propionic acid, and butyric acid) in mice treated with 5-FU. The changes in fecal SCFA levels in this study were consistent with shifts in the intestinal microbiome composition. We thus speculated that in addition to promoting β-catenin downregulation, ABP also promotes anti-tumor effects *in vivo* by increasing the abundance of SCFA-producing bacteria and the levels of beneficial SCFAs. Such improvement in the intestinal microbiome and associated metabolite production may, in turn, reverse the 5-FU-induced immunosuppression and dysbiosis.

**FIGURE 10:**
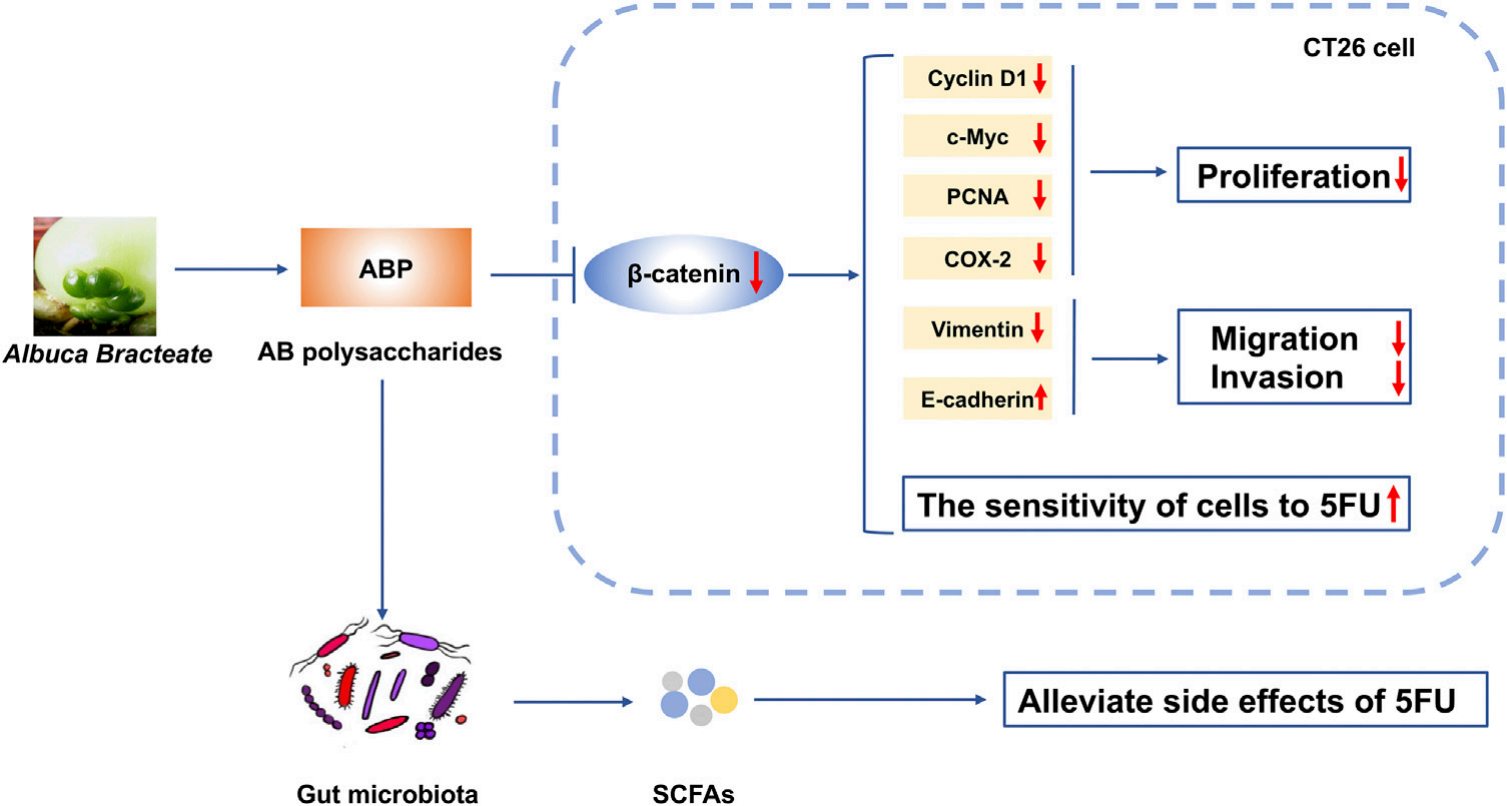
Schematic diagram depicting the mechanism of action underlying the effect of ABP as a regulator of CRC progression. ABP suppresses CRC cell proliferation, migration, and invasion by influencing the Wnt/β-catenin signaling pathway. ABP treatment further increases the sensitivity of CRC cells to 5-FU and thereby decreases the incidence of 5-FU-related adverse effects. ABP, *Albuca Bracteate* polysaccharides; CRC, colorectal cancer.

Together, our data indicate that ABP treatment alone or in combination with 5-FU could reduce the expression of β-catenin and related proteins in CRC cells. ABP treatment synergistically enhances the anti-tumor efficacy of 5-FU by promoting β-Catenin downregulation, improving the composition of intestinal flora, and elevating fecal SCFA levels (Figure 10). The results imply that ABP may be a favorable anti-cancer agent for enhancing the efficacy of chemotherapy.

Our study suggests that ABP is a promising anti-cancer drug with the potential to serve as a valuable chemotherapy adjuvant agent for the clinical treatment of CRC. However, there are some limitations of the study. First, further preclinical research is required to investigate the pharmacologic action of ABP, although our data showed that β-catenin signaling is involved in the anti-tumor effect of ABP. Second, the anti-tumor effects of ABP were only performed in CT26, a murine colon carcinoma cell line. Effects and underlying mechanisms of ABP on human CRC cell lines *in vitro* and *in vivo* models will require intensive research.

## Data Availability

The datasets presented in this study can be found in online repositories. The names of the repository/repositories and accession number(s) can be found in the article/Supplementary Material. The 16S rRNA gene sequence data (accession number: PRJNA733597) have been deposited in the NCBI’s Sequence Read Archive database.
