# Supplementary material for: Albuca Bracteate Polysaccharides Synergistically Enhance the Anti-Tumor Efficacy of 5-Fluorouracil Against Colorectal Cancer by Modulating β-Catenin Signaling and Intestinal Flora
Source: Front Pharmacol. 2021 Sep 3;12:736627. doi: 10.3389/fphar.2021.736627 (PMC8450769; doi:10.3389/fphar.2021.736627)
Supplement: Supplementary file 1 [file DataSheet1.docx]

**Supplementary Figures**

**Figure S1**. The effects of ABP in E-cadherin, c-Myc, Cyclin D1 and COX-2 *mRNA* levels in CT26 cells.

**Figure S2**. Knockdown of β-catenin by *siRNAs* increases the sensitivity of CT26 cells to 5-FU treatment.

**Figure S3**. The synergistic effects between ABP and 5-FU in CT26 cells.

**Figure S1.** The effects of ABP in E-cadherin, c-Myc, Cyclin D1 and COX-2 mRNA levels in CT26 cells. CT26 cells were treated with ABP for 24 h, The total RNA was extracted from the cells using TRIzol (Thermo Fisher Scientific), and the complementary DNA was synthesized using the PrimeScript reverse transcription reagent Kit (Takara, Japan) according to the manufacturer’s protocol. The forward and reverse primers were used, and *mRNA* expressions were detected.

**Figure S2**. Knockdown of β-catenin by *siRNAs* increases the sensitivity of CT26 cells to 5-FU treatment. Mouse *siRNAs* were purchased from the suppliers as indicated and transfected with Lipofectamine 2000 according to the manufacturer’s instructions for 24h, cells were treated with 5-FU for 24h, CCK-8 assay examining the sensitivity of CT26 cells to 5-FU treatment.

**Figure S3.** The synergistic effects between ABP and 5-FU in CT26 cells. **(A)** Synergy scores were calculated using the Synergyfinder software. Loewe and HSA Synergy scores > 10 indicate synergism (red regions) and scores < -10 indicate antagonism (green regions). **(B)** CT26 cells were treated with ABP or 5-FU alone or in combination and cell viability was assessed. A dose-effect analysis of the drug combinations to determine synergism/antagonism based on the Chou-Talalay method was performed using the Compusyn software. Combination index (CI) values shown above the bars were mostly < 1 indicating a synergistic effect of both drugs at the specific concentrations. CI values > 1, indicate antagonism.
